# RNA Helicase A Regulates the Replication of RNA Viruses

**DOI:** 10.3390/v13030361

**Published:** 2021-02-25

**Authors:** Rui-Zhu Shi, Yuan-Qing Pan, Li Xing

**Affiliations:** 1 Institute of Biomedical Sciences, Shanxi University, 92 Wucheng Road, Taiyuan 030006, China; ruizhushi@163.com (R.-Z.S.); pyqing0206@163.com (Y.-Q.P.); 2 Key laboratory of Medical Molecular Cell Biology of Shanxi Province, Shanxi University, Taiyuan 030006, China; 3 Shanxi Provincial Key Laboratory for Prevention and Treatment of Major Infectious Diseases, Shanxi University, Taiyuan 030006, China

**Keywords:** RNA helicase A, RNA virus, transcription, translation, replication

## Abstract

The RNA helicase A (RHA) is a member of DExH-box helicases and characterized by two double-stranded RNA binding domains at the N-terminus. RHA unwinds double-stranded RNA in vitro and is involved in RNA metabolisms in the cell. RHA is also hijacked by a variety of RNA viruses to facilitate virus replication. Herein, this review will provide an overview of the role of RHA in the replication of RNA viruses.

## 1. Introduction

RNA Helicase A (RHA), also known as DHX9 or Nuclear DNA Helicase II (NDH II), is a member of DExH-box family of superfamily 2 (SF2) helicases. RHA plays regulatory roles in a variety of cellular processes including transcription [1], translation [2], RNA splicing [3,4,5], RNA editing [1], RNA transport [6,7], microRNA genesis [8], circular RNA genesis [6,9,10,11], and maintenance of genomic stability [12,13].

**Figure 1:**
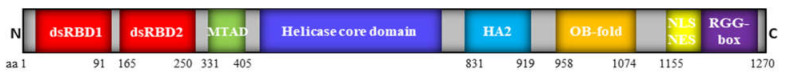
Schematic view of domains of RHA. The numbers represent the positions of RHA amino acid (aa) residues that delineate each functional domain or motif. dsRBD1: double-stranded RNA-binding domain 1; dsRBD2: double-stranded RNA-binding domain 2; MTAD: minimal transactivation domain; HA2: helicase-associated domain 2; OB-fold: oligonucleotide/oligosaccharide-binding fold; NLS/NES: nuclear localization/export signals.

RHA is predominantly retained in the nucleus, but able to shuttle back to the cytoplasm to fulfill its duty [14,15]. Human RHA was first isolated from nuclear extracts of HeLa cells [16]. RHA can unwind duplex RNA possessing a 3’ single-stranded RNA (ssRNA) tail [17] by hydrolyzing nucleoside triphosphates as energy resources [18]. The full-length RHA is a 140-kDa protein composed of 1270 amino acids [19]. RHA contains two double-stranded RNA-binding domains (dsRBDs) at its N-terminus and a helicase core domain in the middle region of the protein [19] (Figure 1). A helicase-associated domain 2 (HA2) is adjacent to the C-terminal end of the helicase core domain [20]. An oligonucleotide/oligosaccharide-binding fold (OB-fold) [21] and a glycine-rich RGG-box are located at the C-terminus of RHA [19]. In addition, RHA has a minimal transactivation domain (MTAD) located in between the second dsRBD (dsRBD2) and helicase core domain [22] and nuclear localization/export signals (NLS/NES) in between OB-fold and RGG-box [22]. 

RNA viruses have an RNA genome. RHA has been found to interact with the RNA or/and proteins of at least 10 RNA viruses from seven families including *Arteriviridae*, *Flaviviridae*, *Hepeviridae*, *Orthomyxoviridae*, *Picornaviridae*, *Retroviridae*, and *Togaviridae*. The involvement of RHA in the replication of a broad variety of RNA viruses may imply that it has potential to serve as an anti-viral target. 

## 2. RHA in Replication of RNA Viruses

### 2.1. Arteriviridae Family: Porcine Reproductive and Respiratory Syndrome Virus (PRRSV)

PRRSV is an enveloped positive-sense RNA virus that belongs to the family *Arteriviridae* [23]. The viral RNA genome is approximately 15 kb in length and contains at least 10 open reading frames (ORFs). The viral nonstructural protein Nsp9 is a core component of the viral replication and transcription complex (RTC) crucial for virus replication [24]. The nucleocapsid (N) protein is responsible for the packaging of viral genome [25].

N protein interacts with Nsp9 to facilitate the production of both viral RNA and infectious progeny viruses [26]. RHA associates with N protein [27,28]. This association can redistribute RHA from the nucleus into the cytoplasmic foci where RTC-mediated virus replication occurs [26]. In addition to viral genomic RNA (gRNA), the replication of PRRSV also produces a set of co-terminal subgenomic mRNAs (sgmRNAs) from which the viral structural proteins are translated [29]. Knockdown of RHA increases the ratio of short sgmRNAs [26]. On the other hand, overexpression of RHA increases the ratio of both longer sgmRNAs and the gRNA and enhances the production of progeny viruses [26]. These results imply that RHA is recruited by the N protein during virus replication to facilitate the synthesis of longer viral RNA [26]. 

### 2.2. Flaviviridae Family: Bovine Viral Diarrhea Virus (BVDV), Classical Swine Fever Virus (CSFV), Dengue Virus (DENV), and Hepatitis C Virus (HCV)

So far, RHA has been found to regulate the replication of at least four viruses from the family *Flaviviridae*. 

#### 2.2.1. BVDV 

BVDV is a member of the Pestivirus genus of the family *Flaviviridae* [30]. BVDV genome is a positive-sense, single-stranded RNA of about 12.3 kb in length and contains one ORF that is flanked by untranslated regions (UTRs) at the 5’ and 3’ ends [31]. UV cross-linking/label transfer method identified that RHA specifically associates with both 3’- and 5’-UTRs. Further biochemical analysis reveals that RHA along with other cellular proteins including NF90/NFAR-1 and NF45 form a complex to establish a functional bridge between 3’- and 5’-UTRs. Depletion of RHA with small interfering RNA (siRNA) significantly reduces the replication of viral RNA [31].

#### 2.2.2. CSFV

CSFV belongs to the *Pestivirus* genus of the family *Flaviviridae*. CSFV infection of pig causes swine fever [32]. 

The genome of CSFV is a positive-sense, single-stranded RNA that contains a single large ORF. The translation of ORF produces a polyprotein that will be further processed by cleavage into a series of smaller mature proteins including structural proteins (C, Erns, E1, E2) and nonstructural proteins (Npro, p7, NS2, NS3, NS4A, NS4B, NS5A, NS5B) [30]. 5’-UTR and 3’-UTR flank the ORF and regulate both the protein translation and the replication of viral RNA [33]. 

Affinity chromatography and UV-crosslinking assays identified that RHA specifically binds to the 3’- and 5’-UTRs [34]. Depletion of RHA in PK-15 cells (porcine kidney cell line) significantly reduces the levels of viral RNA and NS3 protein, and the titers of progeny viruses. The results indicate that RHA is an important cellular factor involved in both the translation and the replication of CSFV [34].

#### 2.2.3. DENV

Dengue virus is a mosquito-borne virus with four major serotypes (DENV1 to DENV4) [35]. Dengue virus infection causes dengue fever, dengue hemorrhagic fever (DHF), or dengue shock syndrome (DSS) [35]. DENV infection is a significant global health concern, particularly for persons living in tropical and subtropical areas. Approximately 100 million cases of dengue virus infection are reported globally per year [36].

The genome of DENV is a positive-sense, single-stranded RNA with 10.7 kb in length and encodes a single large polyprotein precursor of 3391 amino-acid residues [37]. Once the virion enters the host cell, the viral genome RNA is released into the cytoplasm to produce a large polyprotein precursor [37]. The polyprotein is processed by cellular and viral proteases into three structural proteins (C: capsid protein; prM/M: membrane protein; and E: envelope protein) and seven nonstructural proteins (NS1, NS2A, NS2B, NS3, NS4A, NS4B, and NS5) [35]. The NS proteins form a replicase complex in the perinuclear region of the endoplasmic reticulum (ER) to initiate viral RNA synthesis and protein translation [38]. 

DENV genome also contains short noncoding regions at both 5’ and 3’ ends [39]. Both 5′-m7GpppG cap and a conserved 3′-terminal stem loop (3’-SL) [40] are involved in the regulation of viral RNA translation and replication by interacting with cellular and/or viral proteins [41]. Recently, a variety of host factors have been identified to be involved in DENV replication [42,43,44]. RHA binds to DENV 3’-SL and forms a heterodimer with NF90. HF90 is a double-stranded RNA binding domain-containing protein that positively regulates dengue virus replication [45]. DENV infection partially redistributes RHA from the nucleus into the cytoplasm [46]. Knockdown of RHA in three permissive cell lines including A549, MDM (monocyte-derived macrophage), and HepG2 cells significantly reduces the replication of DENV strains NGC and 16681 and other flavivirus *Japanese encephalitis virus* (JEV), but slightly affects the replication of *Zika virus* (ZIKV). RHA does not affect the attaching of DENV virion to the cell, nor virus endocytosis [46]. RHA does not promote viral protein translation, but is required for DENV RNA replication by binding to viral RNA and associating with viral NS1, NS2B3, and NS4B but not NS5. Furthermore, the K417R mutant RHA lacking ATP binding activity works as efficiently as wild-type protein to facilitate virus replication, indicating that the ATPase/helicase activity of RHA is dispensable for the function of RHA in DENV replication [46].

#### 2.2.4. HCV

HCV is the causative pathogen for hepatitis C and some human cancers such as hepatocellular carcinoma (HCC) and lymphomas [47]. HCV is a small enveloped RNA virus. HCV genome is a positive-sense, single-stranded RNA of about 9.7 kb in length and contains only one ORF flanked by UTRs at the 5’- and 3’-ends [48]. 

The 5’-UTR of HCV regulates both the translation and the replication of the viral RNA [48]. In a biotin-tagged RNA pulldown assay, RHA was found to associate with the cognate double-stranded RNA of 5’-UTR [49]. Depletion of RHA reduces both viral RNA synthesis and the levels of viral proteins including NS3 and NS5A, indicating that RHA is an essential cellular protein for HCV replication [50].

### 2.3. Hepeviridae Family: Hepatitis E Virus (HEV)

HEV belongs to the genus *Orthohepevirus* of the family *Hepeviridae*. It is the causative pathogen of Hepatitis E that is characterized by liver inflammation and usually follows an acute and self-limiting course of illness [51]. The viral particle is nonenveloped and contains a positive-sense, single-stranded RNA of about 7.2 kb in length. The viral genome contains three ORFs (ORF1, ORF2, and ORF3) that are flanked by UTRs at the 5’ and 3’ ends [52]. The UTRs of HEV genome function as cis-acting elements to control the initiation of virus replication [53].

RNA affinity chromatography followed by mass spectrometry analysis identified that RHA specifically interacts with the 3’-UTR of HEV in three mammalian cell lines (HepG2/C3A, A549, and Caco2), but the biological significance of these interactions is not known [54].

### 2.4. Orthomyxoviridae Family: Influenza A

*Influenza A* viruses cause acute respiratory disease in humans and a variety of animal species. The infection of human *influenza A* virus can cause seasonal epidemics of influenza [55]. *Influenza A* virus is an enveloped virus with a genome made up of eight negative sense, single-stranded, RNA segments, which encode 11 viral proteins [56]. 

Non-structural protein 1 (NS1) regulates virus replication, viral protein synthesis, host immune responses, and cellular signaling pathways [57]. NS1 complexes with RHA in an RNA-dependent manner during virus replication in A549 cells [58]. Depletion of RHA results in a significant reduction in virus yield, polymerase activity, and the levels of all species of viral RNAs including mRNA, cRNA, and vRNA during virus replication, indicating that RHA participates in both viral RNA transcription and replication. The ATPase-dependent helicase activity is required for the function of RHA in *influenza A* replication [58]. 

### 2.5. Picornaviridae Family: Foot and Mouth Disease Viruses (FMDV)

FMDV belongs to the *Aphthovirus* genus of the family *Picornaviridae* [59]. FMDV infection causes a highly contagious disease for cloven-hoofed animals [60]. The viral particle is a nonenveloped icosahedron containing a positive-sense, single-stranded RNA genome. A single ORF in the RNA genome is flanked by UTRs at the 5’- and 3’- ends. The genomic RNA serves as not only the mRNA to produce a viral polyprotein but also the template for RNA synthesis [61]. 

FMDV replication redistributes RHA from the nucleus to the cytoplasm of infected bovine kidney LFBK cells by suppressing the methylation of RHA [62]. The cytoplasmic RHA associates with FMDV 2C and 3A as well as cellular poly(A) binding protein (PABP) and is in close proximity to the foci where the virus is replicating [62]. In vitro biochemical analysis reveals that RHA binds specifically to the S fragment of FMDV 5’-UTR, suggesting that it may interact with the virus genome in the host cell. The depletion of RHA by siRNA significantly reduces both the titer of progeny viruses and the expression of viral proteins including 3D^pol^. These results indicate that RHA plays an essential role in the replication of FMDV probably by forming ribonucleoprotein complexes with viral and cellular proteins including 2C, 3A, and PABP at the 5’-UTR of viral genome [62]. 

### 2.6. Retroviridae Family: Human Immunodeficiency Virus Type 1 (HIV-1) and Other Retroviruses

HIV-1 is a member of lentivirus of the family *Retroviridae*. HIV-1 infection can cause the acquired immune deficiency syndrome (AIDS) [63]. Upon entry into host cells, the genomic RNA of HIV-1 is reversely transcribed into minus-strand strong-stop cDNA (-sscDNA) followed by conversion into double-stranded DNA that integrates into the host cell chromosomes as a provirus. The transcription of proviral DNA produces a full-length, unspliced viral RNA, which is translated into Gag and GagPol and later encapsidated into the viral particles as viral RNA genome. tRNA^Lys3^ is selectively packaged into viral particles and annealed to viral genomic RNA as a primer in reverse transcription [64]. 

RHA associates with HIV-1 Gag in an RNA-dependent manner and is incorporated into HIV-1 particles (Roy et al., 2006). HIV-1 particles produced from RHA-depleted 293T cells are less infectious and have a reduced ability to generate -sscDNA in newly infected cells. These phenomena are attributed to the reduced annealing of tRNA^Lys3^ to the genomic viral RNA (Roy et al., 2006; Xing et al., 2011) and the compromised processivity of reverse transcription by reverse transcriptase (Brady et al., 2019).

The annealing of tRNA^Lys3^ to viral genomic RNA is initiated by Gag [65] and then fine-tuned by nucleocapsid (NC) protein, a proteolytic derivative of Gag [66]. RHA promotes Gag-medicated annealing of tRNA^Lys3^ by altering viral RNA conformation [67]. Enhancement of tRNA^Lys3^ annealing plausibly benefits from the repetitive nature of unwinding by RHA, because the repetitive unwinding makes the unwound RNA strand more accessible for pairing with the third incoming RNA [17]. 

RHA promotes the transcription of HIV-1, facilitates the export of viral RNAs from the nucleus, and also affects the splicing process. The HIV-1 trans-activation response (TAR) element is a conserved RNA fragment that displays a dynamic hairpin structure [68]. The TAR element serves as the binding site for viral Tat protein and other cellular proteins including the cis-acting transactivation response element-binding protein (TRBP) in in trans activation of HIV-1 long terminal repeat promoter [69]. The TAR element is also a preferred target of dsRBDs of RHA in both in vitro and in vivo assays [70]. Overexpression of wild-type RHA rather than mutant RHA with defect in ATPase activity profoundly enhances viral mRNA synthesis and virion production [70]. RHA stimulates HIV-1 transcription by enhancing the occupancy of RNA polymerase II (RNAP II) on the proviral DNA [21]. The MTAD of RHA is critical to RHA-mediated activation of HIV-1 transcription, since deletions in MTAD significantly reduce the steady-state level of HIV-1 RNA transcripts [71]. 

In addition to full-length, unspliced (∼9.2 kb) viral RNA, HIV-1 transcription also produces singly spliced (∼4.0 kb) or multiply spliced (∼1.8 kb) RNAs [72]. The singly spliced RNAs encode viral Env, Vpu, Vpr, or Vif proteins, whereas multiply spliced RNAs encode Tat, Rev, and Nef proteins. HIV-1 also produces a number of exon 6D-containing spliced viral RNAs encoding chimeric protein Tev (Tat–Env–Rev fusion protein) [71,73,74]. Overexpression of RHA increases the ratio of unspliced and singly spliced RNAs to multiply spliced ones [71,75]. Additionally, mutation analysis shows that OB-fold of RHA can modulate HIV-1 RNA splicing [21]. 

The nuclear export of unspliced or singly spliced HIV-1 RNAs is mediated by the interaction of trans-acting viral protein Rev with the cis-acting Rev response RNA element (RRE) [76]. RHA binds to HIV-1 RRE independently of Rev to promote nuclear export of viral RNAs in a helicase activity-dependent manner [75]. 

RHA promotes the translation of HIV-1 Gag by binding to the posttranscription control element (PCE) in the 5’-UTR of HIV-1 RNA [77]. The ATPase-dependent helicase activity is required for the role of RHA in Gag translation. PCE is located within RU5 region of HIV-1 5’-UTR. PCE is also conserved in the genomes of other retroviruses including *spleen necrosis virus* (SNV) [78,79], *reticuloendotheliosis virus A* (REV-A), *human T-cell leukemia virus type 1* (HTLV-1) [80], *Mason–Pfizer monkey virus* (MPMV) [81], or a set of cellular mRNAs [82]. Studies with HTLV-1 reveal that the interaction of RHA with PCE can promote the polysome association with viral RNA [80]. 

The interaction of RHA with HIV-1 RNA was investigated by performing a genome-wide analysis [83]. 5’-UTR of HIV-1 RNA is the major target for RHA, whereas RRE is the minor target during virus replication. Both dsRBD1 and dsRBD2 of RHA are essential for the binding of RHA to HIV-1 RNA, because deletion of either one leads to undetectable association of RHA with HIV-1 RNA in the cells [83]. Furthermore, the mutant RHA with deletion of either dsRBD1 or dsRBD2 does not promote the in vivo annealing of tRNA^Lys3^ to viral RNA, nor is the mutant RHA packaged into viral particles [83]. Two conserved lysine residues in each dsRBD of RHA, i.e., K54 and K55 in dsRBD1 and K235 and K236 in dsRBD2, are critical to the binding of RHA to HIV-1 RNA during virus replication [84]. 

### 2.7. Togaviridae Family: Chikungunya Virus (CHIKV)

CHIKV belongs to the genus *Alphavirus* of the family *Togaviridae*. The virus is transmitted by mosquitoes. CHIKV infection causes fever, rash, headache, joint and muscle pains, or joint swelling [85]. 

CHIKV genome is a positive-sense, single-stranded RNA of 11.6 kb in length [86]. It contains two ORFs, ORF1 and ORF2, that encode nonstructural and structural proteins, respectively [87]. Upon infection of host cell, the ORF1 is translated directly into P1234 and P123 polyproteins that will be further processed by self-cleavage to produce mature nonstructural proteins (nsP1, nsP2, nsP3, and nsP4) [87]. 

CHIKV infection of human HeLa cells significantly decreases the nuclear level of RHA in the cell and redeploys a fraction of RHA into plasma membrane-proximal cytoplasmic foci where the virus is replicating [88]. Immunoprecipitation experiments reveal that RHA complexes with CHIKV genomic RNA and nsPs in infected cells mainly by direct binding to the hypervariable domain (HVD) of nsp3. However, depletion of RHA with siRNA or clusters of regularly interspaced short palindromic repeats (CRISPR)/Cas9-mediated genome editing significantly enhances viral RNA synthesis. In contrast, RHA depletion dramatically decreases the expression of early nsPs, indicating that RHA is required for the translation of viral proteins. Taken together, RHA plays two distinct roles in CHIKV replication, i.e., inhibiting CHIKV RNA replication but enhancing the translation of the incoming viral genome [88]. 

## 3. Concluding Remarks

The growing evidences show that RHA associates with a broad variety of RNA viruses. In addition to serving as a proviral factor in RNA virus replication, RHA can act as an RNA sensor in the innate immune system. In myeloid dendritic cells (mDCs), RHA interacts with IPS-1 to sense double-stranded RNA in the activation of nuclear factor kappa B (NF-κB) and IFN regulatory factor 3 [89]. Knockdown of RHA dramatically reduced the ability of mDCs to produce IFN-α/β and proinflammatory cytokines in response to infection of influenza A and reovirus [89]. Based on the available results, while the mechanisms by which RHA participates in the replication of different RNA viruses would be diverse, we still can see some characteristics that appear to be common. For example, studies reveal that the helicase activity is often required for the tested functions of RHA during virus replication and RHA always associates with the specific region of viral RNAs, such as 5’- and/or 3’-UTRs that usually adopt complexed secondary structures (Table 1). These features are reminiscent of the findings that RHA preferably binds to the double-stranded RNA rather than single-stranded one and is able to unwind the dsRNA [17,18]. Therefore, it would be plausible to postulate that RHA is employed to remodel the complexed RNA structure by using its helicase activity to regulate virus replication at diverse steps including transcription, translation, RNA synthesis, or tRNA^Lys3^ annealing. However, how the RHA recognizes and then binds to the specific region in the viral RNA has yet to be explored. Several viral proteins have been shown to associate with or bind directly to the RHA during virus replication (Table 1). Those viral proteins may help to hijack RHA and then redirect the RHA to the places where it is needed. To understand the role of RHA in virus replication also requires the insightful information about the interactions of RHA with viral proteins or RNA elements. 

## Figures and Tables

**Table 1:** The role of RHA in the replication of RNA viruses.

Virus	Family	Biological Processes Affected	Helicase Activity Required (+) or not (˗)	Viral Nucleic Acids Involved	Viral Protein Involved
PRRSV	*Arteriviridae*	Genomic replication [26]	?	?	N
BVDV	*Flaviviridae*	Genomic replication [31]	?	3’-UTR, 5’-UTR	?
CSFV	*Flaviviridae*	Translation and genomic replication [34]	?	3’-UTR, 5’-UTR	?
DENV	*Flaviviridae*	Genomic replication [46]	˗	3’-UTR	NS1, NS2B3, NS4B
HCV	*Flaviviridae*	Translation and genomic replication [49,50]	?	5’-UTR	?
HEV	*Hepeviridae*	Unknown [54]	?	3’-UTR	?
Influenza A	*Orthomyxoviridae*	Transcription and genomic replication [58]	+	?	NS1
FMDV	*Picornaviridae*	Translation and genomic replication [62]	?	5’-UTR	2C and 3A
HIV-1	*Retroviridae*	Transcription [70] Translation [77] Reverse transcription [90] Splicing [21] RNA export [75]	+ + + + +	5’-UTR, RRE	Gag
CHIKV	*Togaviridae*	Translation and genomic replication [88]	?	?	Nsp3

?: not determined.

## Data Availability

No new data were created or analyzed in this study. Data sharing is not applicable to this article.

## Abbreviations

BVDV	bovine viral diarrhea virus
CHIKV	Chikungunya virus
CRISPR	clusters of regularly interspaced short palindromic repeats
CSFV	classical swine fever virus
DENV	Dengue virus
dsRBD	double-stranded RNA-binding domain
FMDV	foot and mouth disease viruses
gRNA	genomic RNA
HA2	helicase-associated domain 2
HCC	hepatocellular carcinoma
HCV	hepatitis C virus
HEV	hepatitis E virus
HIV-1	human immunodeficiency virus type 1
HTLV-1	human T-cell leukemia virus type 1
JEV	Japanese encephalitis virus
mDCs	myeloid dendritic cells
MTAD	minimal transactivation domain
NF-κB	nuclear factor kappa B
N protein	nucleocapsid protein
NC	nucleocapsid
NLS/NES	nuclear localization/export signal
NDH II	Nuclear DNA Helicase II
NS1	non-structural protein 1
OB-fold	oligonucleotide/oligosaccharide-binding fold
ORF	open reading frame
PABP	poly(A) binding protein
PRRSV	porcine reproductive and respiratory syndrome virus
RHA	RNA helicase A
RNAP II	RNA polymerase II
RRE	Rev response RNA element
RTC	replication and transcription complex
siRNA	small interfering RNA
SF2	superfamily 2
sgmRNA	subgenomic mRNA
-sscDNA	minus-strand strong-stop cDNA
ssRNA	single-stranded RNA
TAR	trans-activation response
Tev	Tat–Env–Rev fusion protein
UTR	untranslated region
WNV	West Nile virus
YFV	yellow fever virus
ZIKV	Zika virus
